# Predictors for mortality due to acute exacerbation of COPD in primary care: Derivation of a clinical prediction rule in a multicentre cohort study

**DOI:** 10.1080/13814788.2021.1959547

**Published:** 2021-08-06

**Authors:** César Alameda, Ángel Carlos Matía, Verónica Casado

**Affiliations:** a Department of Information Systems and Health Outcomes, Castile-Leon Regional Health Authority, Valladolid, Spain; b Department of Education and Professional Development, Castile-Leon Regional Health Authority, Valladolid, Spain; c Department of Health, Castile-Leon Government, Valladolid, Spain; d Department of Medicine, Dermatology and Toxicology, University of Valladolid, Valladolid, Spain

**Keywords:** Asthma/COPD, prognosis/prognostic research, general practice/family medicine, general, multivariate analysis, incl. modelling

## Abstract

**Background:**

In primary care (PC), 80% of the acute exacerbations of chronic obstructive pulmonary disease (AECOPD) are treated. However, no predictive model has been derived or validated for use in PC to help general practitioners make decisions about these patients.

**Objectives:**

To derive a clinical prediction rule for mortality from any cause 30 days after the last PC visit.

**Methods:**

Between December 2013 and November 2014, we performed a cohort study with people aged 40 and over who were treated for AECOPD in 148 health centres in Spain. We recorded demographic variables, past medical history, signs, and symptoms of the patients and derived a logistic regression model.

**Results:**

In the analysis, 1,696 cases of AECOPD were included and 17 patients (1%) died during follow-up. A clinical prediction rule was derived based on the exacerbations suffered in the last 12 months, age, and heart rate, displaying an area under the receiver operating characteristic curve of 0.792 (95% confidence interval, 0.692–0.891) and good calibration.

**Conclusion:**

This rule stratifies patients into three categories of risk and suggests to the physician a different action for each category: managing low-risk patients in PC, referring high-risk patients to hospitals and taking other criteria into account for decision-making in patients with moderate risk. These findings suggest that it is possible to accurately estimate the risk of death due to AECOPD without complex devices. Future studies on external validation and impact assessment are needed before this prediction rule may be used in clinical practice.

KEY MESSAGES

- In primary care, 80% of COPD exacerbations are attended.
- Short-term death due to COPD exacerbation can be accurately predicted in primary care without any complex instrument.
- Further research is needed to perform an extensive validation study in primary care for this and similar predictive models.

## Introduction

Chronic obstructive pulmonary disease (COPD) is the fourth most frequent cause of death in the world. It is expected that mortality from COPD will continue to worsen in the coming decades, mainly due to the increase in tobacco consumption in low- and middle-income countries [1].

Many exacerbations occur throughout the life of a person with COPD, which the Global Initiative for Chronic Obstructive Lung Disease (GOLD) guidelines define as ‘an acute worsening of respiratory symptoms that result in additional therapy’ [2]. Until recently, the exacerbations were considered accessory phenomena without influence on the disease itself. However, numerous studies have shown that exacerbations contribute decisively to the deterioration of lung function, the quality of life of people with COPD and work productivity, in addition to worsening of the prognosis and the increase in associated costs [2–6].

A subgroup of people with COPD suffer frequent exacerbations: those with two or more exacerbations per year. These people suffer a faster deterioration of lung function, longer time at home, more inferior quality of life, higher probability of hospital admission, and higher risk of death than those with fewer exacerbations, regardless of the degree of deterioration of their lung function [7]. The best predictor of the exacerbation frequency in patients is the number of exacerbations they had in the previous year [8].

Exacerbations of COPD are heterogeneous. Lower respiratory inflammation is different depending on whether the aetiology is viral or bacterial infections. Also, patient characteristics affect the severity of the exacerbation. Decision-making in this context is complex and a tool to help physicians would be useful for both doctors and patients [9].

As expressed in the GOLD guidelines, ‘prevention, early detection, and prompt treatment of exacerbations are vital to reduce the burden of COPD.’ Although some predictive models have been published, they were derived and validated in the hospital setting. Almost all these models include variables that cannot be assessed in primary care (PC) [10–16]. We hypothesised that past medical history, symptoms, and signs in a person who suffers an acute exacerbation of COPD (AECOPD) and is treated in PC allow predicting his or her death in the short term. The objective of this study was to derive a clinical prediction rule (CPR) that contained these predictors and supported making the best decisions in the care provided to these patients.

## Methods

### Study design and participants

Methods have been described in detail elsewhere [17]. We designed a cohort study in PC including all people aged 40 and over who were treated between December 2013 and November 2014 in one of the 150 health centres (HC) of the Spanish provinces of Burgos, Salamanca, Soria, Valladolid, and Zamora and who were diagnosed with AECOPD (code ICD-9-CM 491.21). At the beginning of the study, these provinces had 736,183 inhabitants between 40 and 79 years of age. We excluded individuals who did not have a diagnosis of COPD in their electronic health record (EHR).

To study the prognosis of the AECOPD episode as a whole and not the prognosis of each visit to the general practitioner (GP) that the patient made during the same episode, we considered the visits made in the four weeks after a visit for AECOPD as part of the same episode of AECOPD. For patients who had several values of the same variable during the same episode of AECOPD, we selected the value corresponding to the visit in which the GP established that the patient had a worse general condition. In patients who made several visits, death was determined 30 days after the last visit. In patients who had several exacerbations, each of them was considered as an independent exacerbation.

### Variables and data measurement

The outcome was death from any cause within 30 days after the last visit due to AECOPD. Evaluation of the independent variables was performed without knowing the result of the outcome. Independent variables were, at the time of the visit, sex, age, peripheral arterial oxygen saturation (SpO_2_), systolic blood pressure (SBP), diastolic blood pressure (DBP), heart rate (HR), peripheral temperature, oedema in the legs, confusion, grade of dyspnoea according to the modified dyspnoea scale of the Medical Research Council (mMRC) scale, Charlson comorbidity index, cardiovascular disease, diabetes mellitus, dementia, cancer (except basal cell carcinoma), being included in the home care programme, type of health centre (rural or urban), season in which the episode began, and the number of exacerbations registered in the EHR in the last 12 months. The last body mass index (BMI) value was also included if it had been registered within the last year, and the last percentage between the observed and the expected forced expiratory volume in one second was included if it had been registered in the last two years.

Confusion was defined as drowsiness, stupor, or coma. Cardiovascular disease was defined as heart failure, acute myocardial infarction, cerebrovascular disease, or peripheral arterial disease. The home care programme is offered to people who spend most of their time in bed (those who can only leave with the help of others) and people with significant mobility impairments (preventing them from leaving home, except in exceptional cases) regardless of the cause, provided that the foreseeable duration of this disability exceeds two months.

### Statistical analysis

The descriptive study of the sample was carried out by a table of frequencies for the qualitative variables and a table of medians and interquartile ranges for the continuous variables. In the univariate analysis, the effect of qualitative variables was studied with Fisher's exact test when the expected frequency was less than five in more than 20% of the cells and with the *χ*^2^ test in the rest of the cases. The effect of quantitative variables was studied with the Mann–Whitney *U* test after studying its normality with the Kolmogorov-Smirnov and Shapiro–Wilk tests in both individuals who died and those who did not.

We carried out descriptive and pattern analyses of the missing data in which all the independent variables and outcomes were included. Assuming that missing data were missing at random, we performed a multiple imputation procedure in which the outcome and all the independent variables were included, using a fully conditional specification method with ten maximum iterations. Minimum and maximum allowable imputed values were defined for SpO_2_, SBP, DBP, HR, BMI, and peripheral temperature, so that the values of the imputations were biologically plausible. The type of univariate model type used was multinomial logistic regression for categorical variables and linear regression for scale variables. Hundred imputations were made because the proportion of missing data was high. The SPSS syntax used can be found in the supplementary information.

We excluded independent variables whose predictive effect had not been demonstrated in previous studies and was not suspected based on the clinical knowledge of the principal investigator. Sex was excluded based on results of the univariate analysis. For categorical variables, categories with few elements were collapsed to maximise their statistical significance in univariate analysis. For continuous variables, extreme outliers, defined as values more than three times the interquartile range below the first quartile or above the third quartile, were truncated. Quantitative variables were not categorised, and their linear relationship with the logit model of the probability of the outcome was studied using the Box-Tidwell test. Only the number of previous exacerbations did not demonstrate linearity, so we iterated some simple transformations for this variable and root square transformation demonstrated linearity. The presence of collinearity between these variables was also studied through the analysis of principal components. Finally, we studied all the possible interactions of age with the remaining predictors by adding their cross-products as well as the interactions between the independent variables with statistically significant relationships.

We derived a logistic regression model for all-cause mortality at 30 days following a stepwise regression method using the logarithm of the likelihood ratio as a selection criterion. The rule to remain in the model was a *p*-value less than 0.157. A variable was considered a confounding factor when by eliminating it from the model, the regression coefficient of another variable changed by more than 10 percent. The internal validity of the model was studied through a bootstrapping resampling simulating 1,000 samples for each of the 100 imputations. We applied a uniform shrinkage factor for the regression coefficients calculated with bootstrapping and re-estimated the intercept based on the adjusted coefficients.

The model’s discrimination was studied with the area under the receiver operating characteristic curve (AUROC), and the calibration was assessed with the calibration slope and the intercept. These performance measures were pooled parameters calculated from the 100 imputations.

To support the clinician making decisions, the result was returned as a mortality risk category (low, moderate, or high), and a different action was proposed for each category. The thresholds were based on predicted probability quintiles and a decision curve analysis was performed.

All the data were collected through an *ad hoc* form implemented in the EHR and the Spanish National Death Index. Statistical analyses were performed with IBM SPSS Statistics 24 and RStudio 1.4.1106 for Windows.

### Ethics

The study protocol was approved by the Burgos Research Ethics Committee (reference CEIC 1185), the Salamanca Research Ethics Committee, the Soria Research Ethics Committee (reference CEIC 1227), the East Valladolid Research Ethics Committee (reference PI-13-115), the West Valladolid Research Ethics Committee (reference PI-13-115) and the Zamora Research Ethics Committee [18,19].

## Results

### Participants and model development

**Figure 1.**
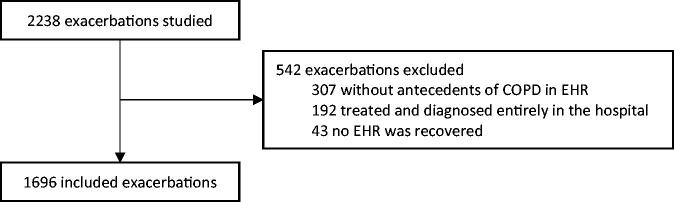
Patient flow diagram. EHR: electronic health record

There were 2,238 exacerbations evaluated in 1,536 people. Of those, 307 (13.7%) exacerbations were excluded because a diagnosis of COPD was not included in the associated EHR, 192 (8.6%) were wholly diagnosed and treated in a hospital, and 43 (1.9%) had no available EHR. Finally, 1,696 exacerbations in 1,054 people (1.6 exacerbations per person) from 148 HC were included in the analysis (Figure 1).

The mean age of participants was 76 years and 84% was male, 17 people (1%) died within 30 days after the last PC visit for AECOPD. Of these participants, 15 (88.2%) died due to AECOPD and one died due to end-stage renal disease; in one case, the cause of death could not be recovered. Complete characteristics of the sample and the relationship of the predictors with the outcome in the univariate analysis are presented in Table 1.

**Table 1.** Descriptive characteristics of the sample and their relation to 30-day mortality in the univariate analysis.

Variable (unit)	*n* (%)^a^		Missing data (%)		OR (CI 95%)		*p*-Value	PC+	PC-
Sex, male	1.419	(83.7)	–		1.46	(0.34 − 6.37)	1	1.06	0.72
Age (years)^b^	78	(69 − 83)	–		1.06	(1 − 1.12)	0.048	–	–
Partial saturation of O_2_ (%)^b^	93	(90 − 95)	683	(40.3)	0.88	(0.84 − 0.93)	<0.001	–	–
Respiratory rate (min^-1^)^b^	20	(15 − 24)	1.596	(94.1)	1.12	(1 − 1.26)	0.043	–	–
Systolic blood pressure (mm Hg)^b^	134.5	(120 − 145)	1.302	(76.8)	0.97	(0.95 − 1)	0.072	–	–
Diastolic blood pressure (mm Hg)^b^	70	(64 − 80)	1.304	(76.9)	0.96	(0.92 − 1.01)	0.136	–	–
Heart rate (min^-1^)^b^	84	(72 − 95)	1.023	(60.3)	1.03	(1.01 − 1.06)	0.014	–	–
Temperature (°C)^b^	36.4	(35.9 − 37.2)	1.433	(84.5)	1.04	(0.5 − 2.16)	0.924	–	–
Oedema	93	(5.5)	1.410	(83.1)	1.77	(0.53 − 5.96)	0.345	1.42	0.8
Retractions or use of accessory respiratory muscles	46	(2.7)	1.551	(91.5)	3.38	(0.55 − 20.99)	0.327	1.95	0.58
Confusion	8	(0.5)	479	(28.2)	30.7	(5.65 − 166.37)	0.004	26.31	0.87
Degree of dyspnoea (mMRC)	–		1.444	(85.1)	–		–	–	–
Dyspnoea grade 0	48	(2.8)	–		–		–	–	–
Dyspnoea grade 1	11	(0.6)	–		–		–	–	–
Dyspnoea grade 2	38	(2.2)	–		–		–	–	–
Dyspnoea grade 3	42	(2.5)	–		–		–	–	–
Dyspnoea grade 4	113	(6.7)	–		9.11	(1.1 − 75.21)^c^	0.024	2.04	0.22
%FEV_1_*	56.4	(48.3 − 68.7)	1.672	(98.6)	–		–	–	–
Body mass index (kg/m^2^)^b^	28.3	(25.6 − 31.6)	739	(43.6)	0.9	(0.75 − 1.09)	0.283	–	–
Exacerbations in the last 12 months^b^	1	(0 − 2)	–		1.57	(1.2 − 2.05)	0.001		
Charlson Index^b^	1	(1 − 2)	–		1.08	(0.88 − 1.33)	0.478	–	–
Charlson Index >1	842	(49.6)	–		2.45	(0.86 − 7)	0.083	1.43	0.58
Cardiovascular disease	363	(21.4)	–		3.31	(1.27 − 8.65)	0.016	2.23	0.67
Peripheral arterial disease	123	(7.3)	–		0.8	(0.1 − 6.06)	1	0.81	1.01
Heart failure	115	(6.8)	–		4.35	(1.39 − 13.55)	0.024	3.56	0.82
Cerebrovascular disease	102	(6)	–		2.1	(0.47 − 9.33)	0.273	1.98	0.94
Acute myocardial infarction	79	(4.7)	–		4.52	(1.27 − 16.06)	0.041	3.9	0.86
Diabetes mellitus	400	(23.6)	–		1	(0.32 − 3.07)	1	1	1
Dementia	24	(1.4)	–		–		1	–	–
Cancer^d^	162	(9.6)	–		2.05	(0.58 − 7.2)	0.217	1.86	0.91
Home care programme	110	(6.5)	–		1.94	(0.44 − 8.59)	0.303	1.83	0.94
Rural health centre	773	(46.6)	–		2.21	(0.81 − 5.99)	0.142	1.43	0.65
Season	–		–		–		–	–	–
Spring	390	(23.0)	–		1.84	(0.68 − 5.01)^e^	0.246	1.54	0.84
Summer	236	(13.9)	–		1.33	(0.38 − 4.66)^e^	0.721	1.27	0.96
Autumn	520	(30.7)	–		0.94	(0.33 − 2.69)^e^	1	0.96	1.02
Winter	550	(32.4)	–		0.44	(0.13 − 1.55)^e^	0.297	0.54	1.22
Death at 30 days	17	(1)	–		–		–	–	–

CI: confidence interval; PC+: positive likelihood ratio; PC-: negative likelihood ratio; O_2:_ oxygen; min: minute; mmHg: millimetres of mercury; °C: degrees Celsius, %FEV_1_: percentage of forced expiratory volume in the first second in relation to the expected FEV; mMRC: modified dyspnoea scale of the Medical Research Council.

a if not otherwise specified.

b Median (interquartile range).

c dyspnoea = 4 vs. dyspnoea <4.

d except basal cell carcinoma.

e vs. rest of stations.

In the pattern analysis, we observed that the variables with the most missing data were those related to the physical examination and the dyspnoea grade. In the descriptive analysis, we observed that the distribution of the missing data in all the variables that had them was related to some of the other variables.

Diastolic blood pressure, peripheral temperature, oedema in the legs, diabetes mellitus, cancer, dementia, type of HC, and season in which the episode began were excluded because they did not demonstrate predictive ability in previous studies and were not suspected according to the clinical criteria of the authors. The categories of the variable ‘grade of dyspnoea according to the mMRC scale’ were combined to form a dichotomous variable called ‘dyspnoea grade 4 according to the mMRC scale.’ Likewise, the Charlson index categories were combined to form a dichotomous variable called ‘Charlson index greater than 1.’ All extreme outliers were considered biologically plausible,17 peripheral arterial oxygen saturation (SpO_2_) values below 75% and four BMI values above 49.74 kg/m^2^ were truncated. A lack of linearity was detected for the variable ‘exacerbations in the last 12 months,’ which was corrected by transforming it into its square root. No significant interactions were found between the predictors. A shrinkage factor of 0.921 was applied.

### Model specification and performance

The regression coefficients for the full model, the variables eliminated, the values of the adjustment statistic in each step, and the results of the internal validation are shown in the supplementary information.

After shrinkage, the equation of the final model is:

P=1/(1+e^(12.156 − 1.127×√x − 0.055×y − 0.03×z)) 

Where: *P* is probability of death from any cause within 30 days after the last primary care visit due to AECOPD; *x* are exacerbations in the last 12 months; *y* is age, measured in years; *z* is heart rate, measured in min-^1^

**Figure 2.**
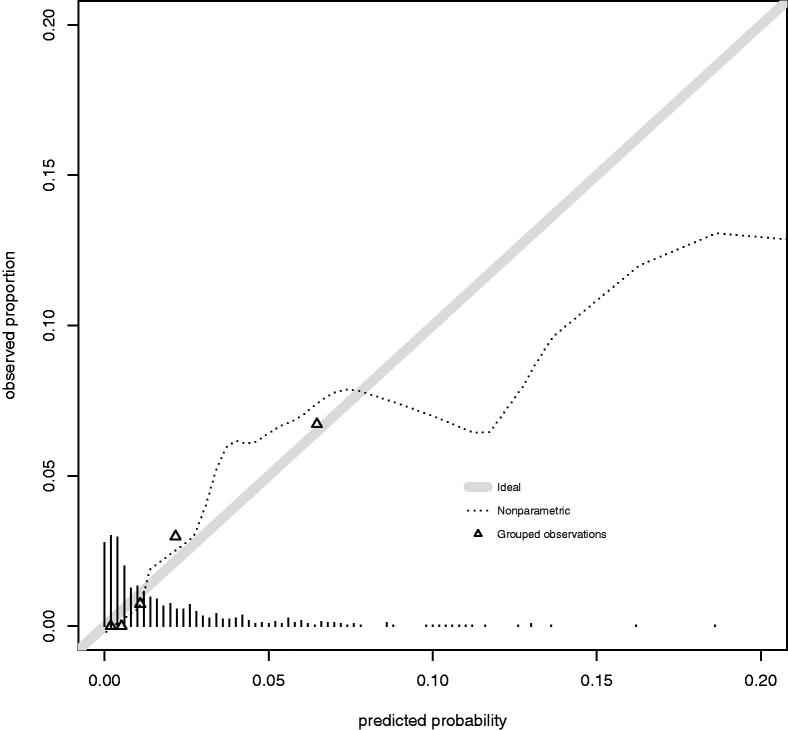
Calibration plot. Solid line represents a model with perfect calibration. Dashed line represents a non-parametric smooth curve for the relation between observed frequency and predicted probability. Triangles are based on quintiles of patients with similar predicted probabilities. Vertical lines above the x axis represents the distribution of predicted probabilities.

**Figure 3.**
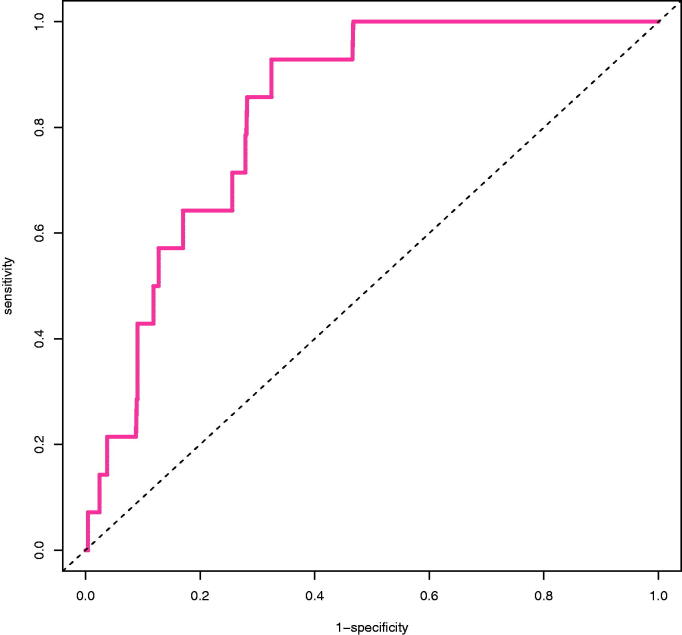
Receiver operating characteristic curve. Dashed line is the no discrimination line (AUROC: 0.5). Solid line represents the model developed (AUROC: 0.811 (95% CI, 0.72–0.902)).

Figure 2 shows the calibration plot of the final model. Calibration slope was 1 (95% confidence interval, 0.45 − 1.55) and intercept was 0 (95% confidence interval, −0.48 − 0.48). AUROC was 0.811 (95% confidence interval, 0.72 − 0.902) for the final model (Figure 3).

This final model proposes three risk categories to be used in clinical practice, based on predicted probability quintiles. Patients with low risk would have a probability of death below the second quintile. Probability of death in these patients is below prevalence so physicians might treat them in primary care. On the other hand, patients in the top quintile of predicted probability might be followed very closely or referred to the hospital. Details about these risk categories are summarised in Table 2.

**Table 2.** Characteristics of each risk category and associated recommendation.

Risk category	Predicted probability	LR+	LR-	Net benefit	Proportion of individuals (%)	Proposed action
Low	<0.008	2.51	0.19	0.0035	40	Probability of death is below prevalence. Consider treating the patient in primary care
Medium	0.008 − 0.029	–	–		20	Probability of death is higher than prevalence. If you decide to treat the patient in primary care, follow him/her closely
High	≥0.03	5.32	0.91	0.0077	20	Probability of death is in the top quintile. Consider referring the patient to the hospital

LR+: positive likelihood ratio; LR-: negative likelihood ratio.

**Figure 4.**
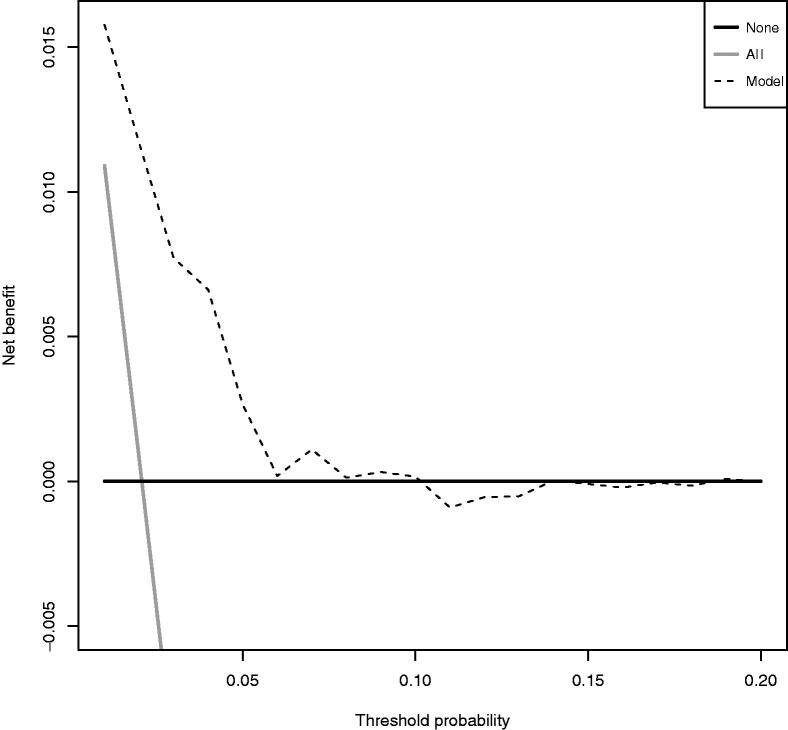
Decision curve. Solid black line represents ‘no intervention’ (derive all). Solid grey line represents the net benefit for ‘intervention in all’ (derive none). Dashed line represents the net benefit for the model.

The decision curve analysis shows that the net benefit of the model is better than alternatives across this range of probabilities (Figure 4).

We recommend estimating the risk of the patient with the equation. As it is not yet implemented in any medical calculator, we propose a simple score system that can be easily remembered and used at the office (Table 3).

**Table 3.** EXAGGERATE score.

Predictor	Points
Exacerbations in the last 12 months	1 point/exacerbation
Age >75 years	1 point
Heart rate >100 min^-1^	1 point
Risk category	Score
Low	0 − 1 points
Medium	2 − 3 points
High	≥4 points

## Discussion

### Main findings

This study derived a CPR for short-term mortality due to an AECOPD treated in PC, based on data collected prospectively in 148 HC over one full year. The predictors are the EXacerbations suffered in the last 12 months of age, the AGE, and the heart RATE (mnemonic, EXAGGERATE), which do not need any complex instrument to be measured. The CPR stratifies patients into three categories of risk based on predicted probability quintiles and suggests to the doctor a different action for each category. Patients with low risk might be followed in primary care. Patients with medium risk might be followed closely if the doctor decides to treat them in primary care. Patients with high risk might be followed very closely or referred to the hospital.

### Strengths and limitations

Age clearly showed a predictive effect in this study; the higher the age of an adult suffering from an acute illness was, the greater their probability of dying in the short term. This finding is also compatible with that of previous studies on the subject [10–16]. The predictive effect of the number of previous exacerbations was also compatible with that in previous studies; as stated in the introduction of the article, patients with frequent exacerbations have a higher risk of death than those with fewer exacerbations [7]. The heart rate had already been shown to have a predictive effect on mortality 30 days after hospital admission due to AECOPD in the derivation and validation of the BAP-65 rule [15], and an acute increase in the partial pressure of carbon dioxide (pCO_2_) had also been shown to interact in a very complex manner with different systems; in cases of moderate acute hypercapnia, an increase in heart rate is often observed. Tachycardia may therefore be an early sign of an acute increase in pCO_2_ prior to the onset of headache, agitation, or a decreased level of consciousness [20].

The study’s main limitation is that our sample size was smaller than required [21]. This situation increases the risk that the model is overfitted; that is, it has a very good predictive performance in the derivation sample and bad predictive performance in new subjects. This risk of overfitting has been reduced by selecting predictors based on external information from the literature review and the authors’ expertise and by using a *p*-value less than 0.157 as the stop rule to exclude a predictor from the model instead of using a value less than 0.05. Despite all the above, the only way to determine the true degree of overfitting in the model presented in this work will be through an external validation study [19].

This study’s other significant limitation is the large proportion of missing data, which may have occurred because the study did not intend to change the way the doctors collected information but took advantage of data collected in the EHR from usual clinical practice. To compensate for power loss that this causes, a multiple imputation procedure was applied in patients with many imputations [22].

Only 16% of the people studied were women, and only two deaths were observed among them, which could reduce the predictive performance of the rule in women. This small proportion of women is because they represent only between 22 and 29% of people with COPD in Spain [23].

Concerns may arise using COPD diagnoses from EHRs. Previous validation studies suggest that such diagnoses have minor sensitivity and high specificity so they should not be used in prevalence studies but may be used to study risk factors [24–26].

As this CPR has not been externally validated, this could only be used in similar patients to its derived ones [27].

### Comparison with existing literature

None of the similar studies published to date derived or validated its rule in PC, where 80% of AECOPD cases are treated [28]. The rules most identical to ours include DeCOPD [12], DECAF [10,16], and the one derived by Esteban and others [11] because the outcome was not intra-hospital mortality but mortality within 30 days after contact with a doctor, such as during hospital emergencies or hospital admission. Likewise, these studies are also based on data obtained from the ‘real world,’ with patients recruited opportunistically while trying to interfere as little as possible in the usual practice of doctors. All these studies had small sample sizes, the continuous variables became categorical (risk of loss of information quality), and the predictors were selected according to the results of the univariate analysis (risk of excluding confounding factors or relevant interaction terms) [29]. In these studies, mortality was high, between 3.5 and 10.4%, because people treated in hospitals usually have more advanced disease and/or a more severe acute episode than people treated in PC.

Discrimination for predicting death from any cause within 30 days after the last visit due to AECOPD in PC in our study was 0,74 (95% confidence interval, 0.571 − 0.909) for the simplified B-AE-D index, a validated, independent of lung function long-term mortality index in COPD [30].

## Conclusion

This is the first predictive model derived from PC for the risk of short-term death due to AECOPD. Although it is a rare event, it can be accurately predicted from knowing the exacerbations suffered in the last 12 months, age, and heart rate. In addition, the rule suggests a different action depending on the calculated risk.

There is enough evidence to design a large validation study in primary care of all existing predictive models. Subsequently, the impact that the best predictive model would have on the results of patient-oriented care compared to usual practices should be studied [27].

## Supplementary Material

TRIPOD Checklist: Prediction Model Development

Supplemental Material

Supplemental Material
