# Supplementary material for: Predictors for mortality due to acute exacerbation of COPD in primary care: Derivation of a clinical prediction rule in a multicentre cohort study
Source: Eur J Gen Pract. 2021 Aug 6;27(1):211–20. doi: 10.1080/13814788.2021.1959547 (PMC8354163; doi:10.1080/13814788.2021.1959547)
Supplement: Supplemental Material [file IGEN_A_1959547_SM7560.docx]

**Supplementary information**

**SPSS syntax for the development of the multiple imputation model**

MULTIPLE IMPUTATION death sex season aecopd_12m saturation sbp dbp hr temperature oedema confusion age bmi rural home_care Charlson ami heart_failure pad dementia diabetes cbd cancer dyspnoea_mMRC

/ IMPUTE METHOD = AUTO NIMPUTATIONS = 100 MAXPCTMISSING = 90 MAXCASEDRAWS = 150 MAXPARAMDRAWS = 2 MAXMODELPARAM = 200

/ CONSTRAINTS death (ROLE = IND)

/ CONSTRAINTS sex (ROLE = IND)

/ CONSTRAINTS season (ROLE = IND)

/ CONSTRAINTS aecopd_12m (ROLE = IND)

/ CONSTRAINTS saturation (MIN = 75.0 MAX = 100.0 RND = 1.0)

/ CONSTRAINTS sbp (MIN = 74.0 MAX = 210.0 RND = 1.0)

/ CONSTRAINTS dbp (MIN = 30.0 MAX = 103.0 RND = 1.0)

/ CONSTRAINTS hr (MIN = 38.0 MAX = 155.0 RND = 1.0)

/ CONSTRAINTS temperature (MIN = 34.1 MAX = 39.4 RND = 1.0)

/ CONSTRAINTS age (ROLE = IND)

/ CONSTRAINTS bmi (MIN = 15.6 MAX = 49.74 RND = 0.01)

/ CONSTRAINTS rural (ROLE = IND)

/ CONSTRAINTS home_care (ROLE = IND)

/ CONSTRAINTS Charlson (ROLE = IND)

/ CONSTRAINTS ami (ROLE = IND)

/ CONSTRAINTS heart_failure (ROLE = IND)

/ CONSTRAINTS pad (ROLE = IND)

/ CONSTRAINTS dementia (ROLE = IND)

/ CONSTRAINTS diabetes (ROLE = IND)

/ CONSTRAINTS cbd (ROLE = IND)

/ CONSTRAINTS cancer (ROLE = IND)

/ CONSTRAINTS dyspnoea_mMRC (ROLE = IND)

/ MISSINGSUMMARIES NONE

/ IMPUTATIONSUMMARIES MODELS

/ OUTFILE IMPUTATIONS=MultipleImputation.

**Regression coefficients of the independent variables in the univariate analysis, the full model with complete cases and the full model after the multiple imputation procedure**

|  |  |  | | **Full models** | | | |
| --- | --- | --- | --- | --- | --- | --- | --- |
|  | **Univariate analysis** | | | **Complete cases***  **N = 59** | | **Multiple imputation***  **N = 1696** | |
| **Variable (unit)** | **N** | **Coefficient (95% CI)** | | **Coefficient (95% CI)** | | **Coefficient (95% CI)** | |
| Age (years) | 1696 | 0.058 | (-0.002 – 0.118) | 0.05 | (-2222 – 2222) | 0.042 | (-0.031 – 0.115) |
| SpO_2_ (%) | 1013 | -0.125 | (-0.179 – -0.071) | 2.99 | (-1525 – 1531) | -0.155 | (-0.261 – -0.049) |
| Respiratory rate (min^-1^) | 100 | 0.118 | (0.002 – 0.234) | - | | - | |
| Systolic blood pressure (mm Hg) | 394 | -0.027 | (-0.057 – 0.003) | -1.404 | (-536 – 533) | -0.023 | (-0.052 – 0.006) |
| Heart rate (min^-1^) | 673 | 0.034 | (0.006 – 0.062) | 0.963 | (-394 – 396) | 0.019 | (-0.014 – 0.052) |
| Retractions or use of accessory respiratory muscles | 145 | 1.219 | (-0.643 – 3.081) | - | | - | |
| Confusion | 1217 | 3.423 | (1.697 – 5.149) | 69.881 | (-121108 – 121248) | 0.432 | (-1.491 – 2.355) |
| Dyspnoea = 4 (mMRC) | 252 | 2.21 | (0.056 – 4.364) | -2.2 | (-56559 – 56555) | 0.226 | (-1.843 – 2.296) |
| Body mass index (kg/m^2^) | 957 | -0.103 | (-0.295 – 0.089) | 1.272 | (-1026 – 1029) | -0.092 | (-0.294 – 0.11) |
| Exacerbations in the last 12 months | 1696 | 0.452 | (0.178 – 0.726) | 31.618 | (-27364 – 27427) | 1.292 | (0.374 – 2.209) |
| Charlson Index > 1 | 1696 | 0.898 | (-0.172 – 1.968) | 8.286 | (-57937 – 57954) | -0.028 | (-1.44 – 1.496) |
| Cardiovascular disease | 1696 | 1.199 | (0.219 – 2.179) | -17.249 | (-30742 – 30707) | 1.069 | (-0.311 – 2.449) |
| Home care programme | 1696 | 0.662 | (-0.856 – 2.18) | -52.061 | (-45188 – 45084) | -0.494 | (-2.309 – 1.321) |
| Intercept |  |  |  | 41.299 | (-84478 – 84561) | -3.495 | (-10.478 – 3.488) |

Respiratory rate and retractions are not included because there are no complete cases for these variables

*O_2_* oxygen, *min* minute, *mm Hg* millimetres of mercury, *mMRC* modified dyspnoea scale of the Medical Research Council

**Steps in the derivation of the final model**

| **Step** | **Eliminated variable** | **-2 Log likelihood** | **Degrees of freedom** | **Nagelkerke’s R^2^** |
| --- | --- | --- | --- | --- |
| 1 | - | 139.354 | 11 | 0.279 |
| 2 | Dyspnoea = 4 (mMRC) | 141.549 | 10 | 0.267 |
| 3 | Confusion | 142.256 | 9 | 0.263 |
| 4 | Home care programme | 142.571 | 8 | 0.261 |
| 5 | Charlson Index > 1 | 142.608 | 7 | 0.261 |
| 6 | Body mass index | 146.365 | 6 | 0.241 |
| 7 | Systolic blood pressure | 151.189 | 5 | 0.215 |
| 8 | Cardiovascular disease | 154.92 | 4 | 0.195 |
| 9 | SpO_2_ | 168.928 | 3 | 0.118 |

*mMRC* modified dyspnoea scale of the Medical Research Council, *SpO_2_* peripheral arterial oxygen saturation

**Results of the simulation by bootstrap sampling**

| **Variable (unit)** | **Regression coefficient** | **Mean regression coefficients of the bootstrap samples** | **BCa 95% CI**^a^ |
| --- | --- | --- | --- |
| Age (years) | 0.059 | 0.057 | (0.019 – 0.109) |
| Heart rate (min^-1^) | 0.032 | 0.033 | (0 – 0.063) |
| Square root of exacerbations in the last 12 months | 1.223 | 1.185 | (0.498 – 2.072) |
| Intercept | -13.605 | -13.3 | (-17.456 – -10.781) |

*min* minute

^a^ Bias-corrected and accelerated 95% confidence interval
